# First‐ever satellite tracking of Black Terns (*Chlidonias niger*): Insights into home range and habitat selection

**DOI:** 10.1002/ece3.10716

**Published:** 2023-11-15

**Authors:** Ann E. McKellar, Sarah J. Clements

**Affiliations:** ^1^ Environment and Climate Change Canada Wildlife Research Division Saskatoon Saskatchewan Canada; ^2^ Department of Biology University of Saskatchewan Saskatoon Saskatchewan Canada; ^3^ School of Natural Resources University of Missouri Columbia Missouri USA; ^4^ Department of Wildlife, Fisheries, & Conservation Biology, Nutting Hall University of Maine Orono Maine USA

**Keywords:** Black Tern, habitat selection, home range, movement tracking

## Abstract

Understanding animal movement across the annual cycle is critical for developing appropriate conservation plans, but the large size and high cost of tracking devices can limit the spatial and temporal resolution at which movement data can be collected, especially for small avian species. Furthermore, for species with low breeding site fidelity, the ability to obtain tracking data from small, archival tags is hindered by low recapture rates. We deployed satellite tracking devices on four adult Black Terns (*Chlidonias niger*), a declining waterbird with low site fidelity, to examine space use and selection of resources within individual breeding home ranges. We also provide a preliminary assessment of habitat use during fall stopover. We found that home ranges were extensive (mean 283.7 km^2^) and distances travelled from the nest substantially larger (up to 35 km) than previously thought (~2.5 km). Terns showed selection for wetlands and open water on the breeding grounds, but also showed selection for developed areas. This may reflect humans selecting similar landscape features for recreation and development as terns, and suggests that terns can tolerate the light degree of development (e.g. cottages, boat launches, etc.) within our study area. Despite a small sample size, this is the first study to track individual Black Terns at a high resolution with implications for conservation and wetland management practices relevant to the spatial scales at which habitat is used by the species.

## Graphical abstract

Using satellite telemetry for the first time to track the movements of this declining waterbird, we show that Black Terns have much larger area requirements during the breeding season than previously thought. Furthermore, individual terns showed strong positive selection for wetlands and open water as predicted, and we present evidence that they tend to avoid cropland but are able to tolerate the light degree of human encroachment present at our study areas (e.g., cottages, boat launches), indicating the importance of maintaining sufficient wetlands within agriculturally‐dominated landscapes.

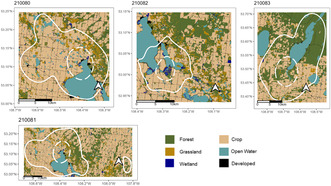

## 1 INTRODUCTION

Habitat loss and degradation are the primary drivers of species imperilment (Newbold et al., 2015; Venter et al., 2006). Understanding how animals move and select habitat features within their ranges can help guide the development of appropriate conservation and management plans (Cañadas et al., 2005; Chetkiewicz & Boyce, 2009). This is especially crucial for migratory birds, which may experience few barriers to movement as they travel vast distances and occupy highly variable habitats across their annual cycle (Anderson et al., 2019; Tonra et al., 2019). Habitat selection, which is the disproportionate use of available resources, can vary both spatially and temporally (Mayor et al., 2009). Animals may select for different habitat features at different spatial scales and at different times of the annual cycle, making the choice of scale all the more important in conservation planning.

While the establishment of large and pristine protected areas has traditionally been the staple or gold standard of biodiversity conservation (Pringle, 2017; Watson et al., 2014), it is becoming increasingly recognized that successful conservation on a global scale will also necessitate the integration of wildlife habitat needs into human‐dominated ecosystems (Kremen & Merenlender, 2018; Perfecto et al., 2009; Tscharntke et al., 2012). The Prairie Pothole Region (PPR) of North America is an intensively altered landscape that has been primarily converted for agricultural use, with the majority of its grasslands and wetlands lost since European settlement and loss rates that continue today. This wetland–grassland ecosystem is also one of the richest and most biodiverse regions in the world (Doherty et al., 2018). Not only is it estimated that the region hosts 50–80% of the continental breeding waterfowl population each year (Batt et al., 1989), it forms the core of the range and area of greatest abundance and density of several other waterbird species, such as Franklin's Gulls (*Leucophaeus pipixcan*) and Black Terns (*Chlidonias niger*) (Beyersbergen et al., 2004). Regional conservation planning requires an understanding of the area and resource requirements of the species within these working landscapes.

The Black Tern is a declining waterbird with the core of its breeding range within the PPR (Heath et al., 2020), and previous work has suggested that it is an area‐dependent species, with wetland size being an important predictor of site occupancy (Brown & Dinsmore, 1986; Naugle et al., 1999). Numerous large‐scale habitat suitability studies on the breeding grounds have documented the importance of both local (e.g. wetland area, wetland type, amount of emergent vegetation at the wetland) and landscape‐level (e.g. forageable area, agricultural intensity within surrounding area) habitat features for the species (Naugle et al., 2000; Shealer & Alexander, 2013; Steen & Powell, 2012; Wyman & Cuthbert, 2016). However, studies differ in terms of which scale is concluded as being most important, and some specific habitat features have been established as important in some studies but not others (e.g. amount of human encroachment; Shealer & Alexander, 2013; Shephard, Reudink, & McKellar, 2023). Interestingly, site occupancy remains very low even at sites predicted to be highly suitable (e.g. <50% Shealer & Alexander, 2013; <20% Wyman & Cuthbert, 2016), which could be an indication that the appropriate scale or features being selected by individuals are not being adequately captured. Most previous work has not distinguished between foraging and nesting habitat needs and has not taken density into account (but see Steen & Powell, 2012), and to date no studies have examined the selection of resources at the individual level within a breeding home range. Nor have any studies documented stopover habitat use in any detail (Heath et al., 2020). Furthermore, previous authors have based their selection of spatial scale (Steen & Powell, 2012; Wyman & Cuthbert, 2016) on foraging distance values reported from a single thesis conducted at a study site in British Columbia, Canada (Mosher, 1986). Specifically, this early work concluded that mean foraging distance from the nest was 2.4 km, which was based on observations and attempts to follow birds as they travelled between the nesting colony and foraging sites, potentially representing a large underestimate of the true areas being used by individuals due to the difficulty of visually tracking birds in this manner.

Here, we provide the first fine‐scale tracking of individual Black Terns on the breeding grounds in Saskatchewan, Canada, via satellite telemetry. We quantify home range sizes and foraging distances, and describe within‐home range habitat selection. We also provide a preliminary assessment of habitat use during the first stopover on fall migration. Despite a small sample size due to technological limitations and life history features of the species, we provide significant new insights into the area requirements of this species, and the habitat features that they select and avoid within an agricultural landscape in the PPR.

## 2 MATERIALS AND METHODS

**FIGURE 1:**
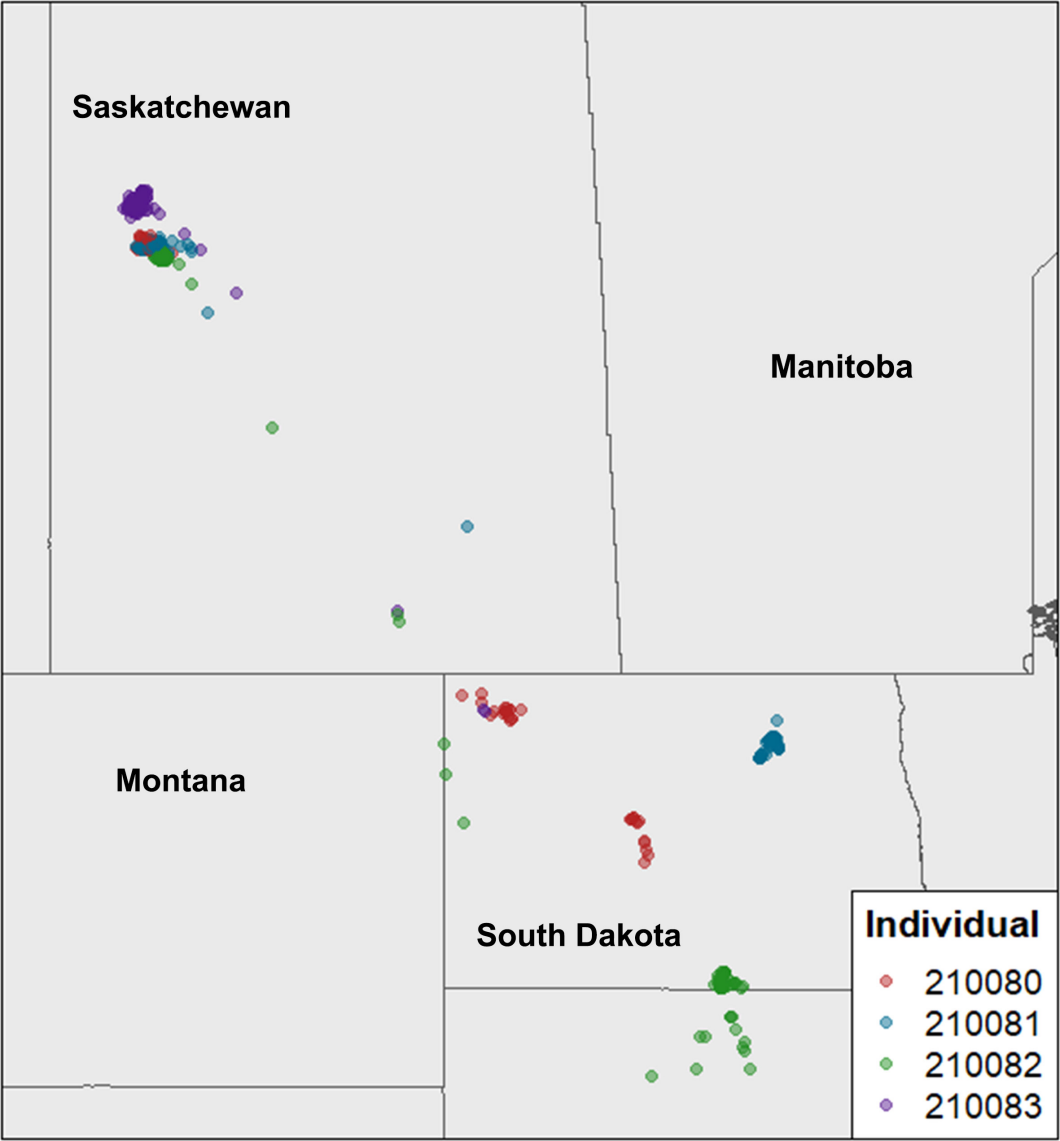
Breeding, migration and stopover locations of four Black Terns tagged with Argos satellite tags at breeding colonies in Saskatchewan, Canada.

Adult Black Terns were captured on the nest during the 2021 breeding season (date range 23 June–3 July) at three colonies in Saskatchewan, Canada (Jackfish Lake: 53.1324, −108.427; Turtle Lake: 53.498, −108.712; Murray Lake: 53.058, −108.326; Figure 1). Capture methods are described in Shephard, Szczys, et al. (2023) and were part of a larger Black Tern banding effort throughout 2018–2022 (AEM, unpubl. data). We banded each individual with a stainless steel Canadian Wildlife Service (CWS) band and took standard morphometrics (mass and tarsus, bill, head‐bill, tail and wing length). We took a drop of blood from the metatarsal vein for molecular sex determination.

Each of four captured adults was equipped with a solar satellite‐transmitting tag (Solar 2 g Argos PTT, Microwave Telemetry) attached with a leg‐loop harness. The leg‐loop harness was a modified version of that described by Mallory and Gilbert (2008) for use on seabirds, and was previously used by Bracey et al. (2020) on Common Terns (*Sterna hirundo*). Total mass of the device and harness did not exceed 3–5% of an individual's body mass. Adult Black Terns at our study site ranged from 50 to 69 g (58.9 ± 3.2 g). To ensure animal welfare guidelines were followed, we restricted tag deployments to individuals weighing more than 62 g. Note that at the time of this study, we were not aware of any satellite‐transmitting tags weighing <2 g, and we required tags to transmit their data (rather than collect and store data, i.e. archival tags such as light‐level geolocators or GPS loggers) due to the low site fidelity of the species (Heath et al., 2020; Shephard, Szczys, et al., 2023). Due to the high cost of tags and the limited availability of heavy‐enough individuals (only ~18% of individuals trapped at our study site weighed more than 62 g; AEM, unpubl. data), we were restricted in our total sample size, and our findings should be considered preliminary until a larger scale, high‐resolution habitat selection study can be conducted.

Individuals appeared to behave normally and flew away after being released following tagging. At least two of the nests (individuals 210080 and 210081), which were part of our long‐term study site at Jackfish Lake, successfully fledged chicks and both tagged birds were observed attending the nest up until the fledge date. Based on the first date each bird travelled more than 30 km between consecutive locations (see below) and visual inspection of movement data, 210080 departed for migration 22 July 2021 and 210081 departed 9 August 2021. We were not able to revisit the other two nests, but 210082 departed for migration 13 July 2021 and 210083 departed 1 August 2021, and up until departure their movement patterns appeared generally consistent with those of 210080 and 210081. Work was conducted under CWS Banding Permit 10431 and Western and Northern Animal Care Permit 21AM02.

Our satellite tags collected and transmitted data opportunistically, without an on–off duty cycle, subject to constraints on battery. In our data set, the mean time between each location and the preceding location was 3.75 h, with 95% of locations collected within 12.75 h of the preceding location and 50% collected within 1.26 h of the preceding location. We uploaded all Argos data from the period June–August 2021 to Movebank (Kranstauber et al., 2011) and applied the Argos Douglas filter (Douglas et al., 2012) upon download. We retained only the three most precise Argos location classes, 1, 2 and 3, for this analysis (accurate to 150–1500 m). We identified breeding and stopover locations based on distance travelled, defining the beginning of migration as the first of at least 3 consecutive directional movements over 30 km each away from the breeding location that led to the eventual arrival (Soriano‐Redondo et al., 2020) at a location in North Dakota or South Dakota (Figure 1). We defined the breeding area based on all data collected before the start of migration, and stopovers as groups of locations within 30 km of one another after the beginning of migration.

We inferred distance travelled for foraging movement based on the straight‐line distance between each known nest location and each location collected by the bird, assuming that all travel away from the nest would be associated with foraging behaviour. While many studies with more frequent duty cycles are able to discern individual foraging trips (e.g. Borrmann et al., 2019), we did not have frequent enough data to characterize individual trips. We then used the filtered data to generate individual home ranges for breeding based on 90% kernel density estimates (KDE) using the amt package (Signer et al., 2019) in Program R (R Core Team, 2022). We chose 90% as the threshold for the home range to ensure we would exclude migration points not accounted for by our distance threshold. We obtained bootstrap confidence intervals using 100 simulations to quantify uncertainty in home range size. We chose to use KDE rather than an autocorrelated KDE (AKDE; Fleming et al., 2015) even though AKDE is recommended for samples close enough in time to be spatially correlated, because the underlying movement model appeared to introduce unrealistic movement patterns that we were not able to account for. We believe this was because there were some time gaps in the data that were long enough to introduce bias to the model, so we felt that the home range estimated by the KDE, although potentially underestimating home range size, was better representative of the ecology of the species.

Then, we used the amt package to simulate 10 random locations for each location collected within the individual's home range (Signer et al., 2019). To obtain land cover information, we used the 2015 Land Cover of North America at 30 Meters data set (CEC et al., 2020), which provides consistent land cover information across Canada, the United States and Mexico. We extracted the percent of land cover within a 150‐m buffer of each point and reclassified land cover classes to include forest, grassland, wetland, developed, crop and open water land cover types. We used a buffer around each location instead of extracting data from each point directly because of the uncertainty in Argos locations. Due to our small sample size, we chose a relatively simple land cover classification scheme that would roughly cover all landscape covariates we thought would be important for tern habitat selection in our study area.

Then, we used a logistic regression to quantify habitat selection using land cover information associated with used and available locations within breeding home ranges. Because we only had four individuals, we investigated third order selection (i.e. within the home range of the animal) and included individual ID in the model as a fixed effect (there were not enough individuals for a random effect). We ran the model using Jags (Plummer, 2003) using the JagsUI (Kellner, 2019) package in Program R (see Appendix S1 for code used to run model). We ran only one model rather than choosing a model selection approach because we had relatively few land cover covariates and aimed to compare effects of each covariate on probability of use rather than find the combination of covariates that most closely predicted probability of use. The model can be written mathematically as follows:

$$\text{logit}\;\left(Y_{i,j}\right)=\beta_{0}+\beta_{1}{\mathrm{FR}}_{i}+\beta_{2}{\mathrm{CR}}_{i}+\beta_{3}{\mathrm{WL}}_{i}+\beta_{4}{\mathrm{WT}}_{i}+\beta_{4}{\mathrm{DV}}_{i}+\beta_{j}{\mathrm{ID}}_{i},$$

where *Y* is probability of use in point *i* for individual *j*. *β*_*0*_ is the intercept, FR, CR, WL, WT, DV and ID are the percent forest, percent cropland, percent wetland, percent open water, percent developed and individual, respectively, associated with each location point *i*. For the individual fixed effect (ID), we used the first individual (ID_1_) as a reference condition. This individual fixed effect accounts for some of the variation not explained by land cover, but it is difficult to interpret ecologically, so we also ran a similar model for each individual bird separately (see Appendix S2), written as follows: logit (*Y*_*i*_) = *β*_0_ + *β*_1_FR_*i*_ + *β*_2_CR_*i*_ + *β*_3_WL_*i*_ + *β*_4_WT_*i*_ + *β*_4_DV_*i*_, where *Y* is probability of use in point *i*, and all other variables are the same as described above. We monitored convergence based on R‐hat <1.1 (Brooks & Gelman, 1998) and visually inspected chains. We present the evidence that an effect was positive or negative as the proportion of posterior samples above or below 0. For all covariates, if more than 85% of posterior samples were above or below zero, we interpret that an effect explained substantial variation in the response (Mosloff et al., 2021). For each covariate, we report the mean effect size (*β*), the 95% credible interval (CI) and the proportion of posterior samples on the same side of 0 as the mean (*P*). We summarized habitat composition by calculating the proportion of each land cover type within a 150‐m buffer of each used location.

## 3 RESULTS

Based on molecular sex determination, all four individuals tagged in this study were males.

### 3.1 Breeding home range size

**FIGURE 2:**
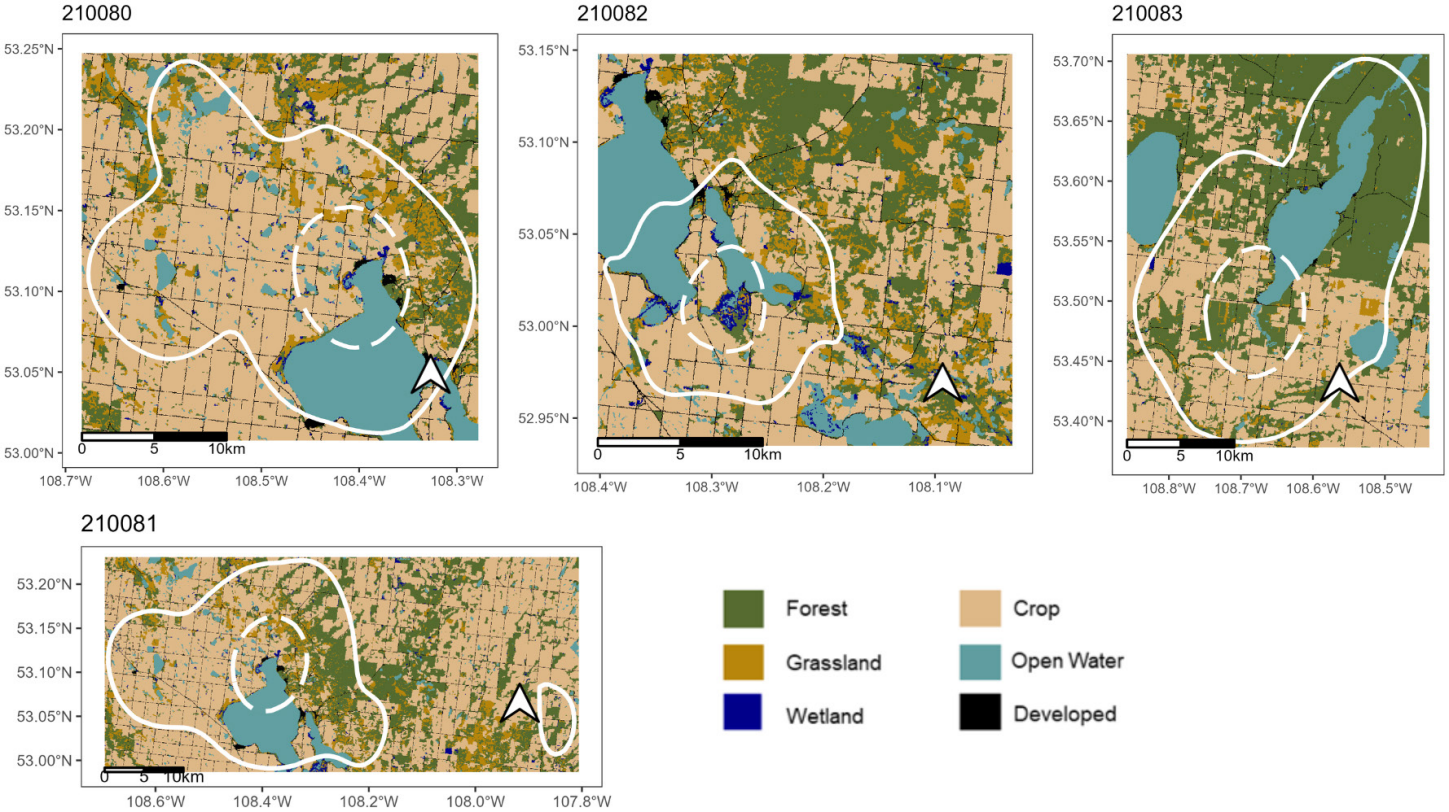
Breeding home ranges of individual Black Terns (*n* = 4). All breeding ranges were located in Saskatchewan, Canada. The solid lines represent the 95% kernel density estimates, the dashed lines represent the 50% kernel density estimates and the base maps are coloured by land cover type.

Based on the 90% KDE, our four individuals had home range sizes of 265.81 (95% CI 199.28, 328.61), 362.60 (95% CI 51.97, 476.79), 95.13 (95% CI 68.51, 118.17) and 411.26 (95% CI 280.79, 515.94) km^2^. The mean area was 283.70 km^2^ (Figure 2).

### 3.2 Distance travelled from nest during breeding season

**FIGURE 3:**
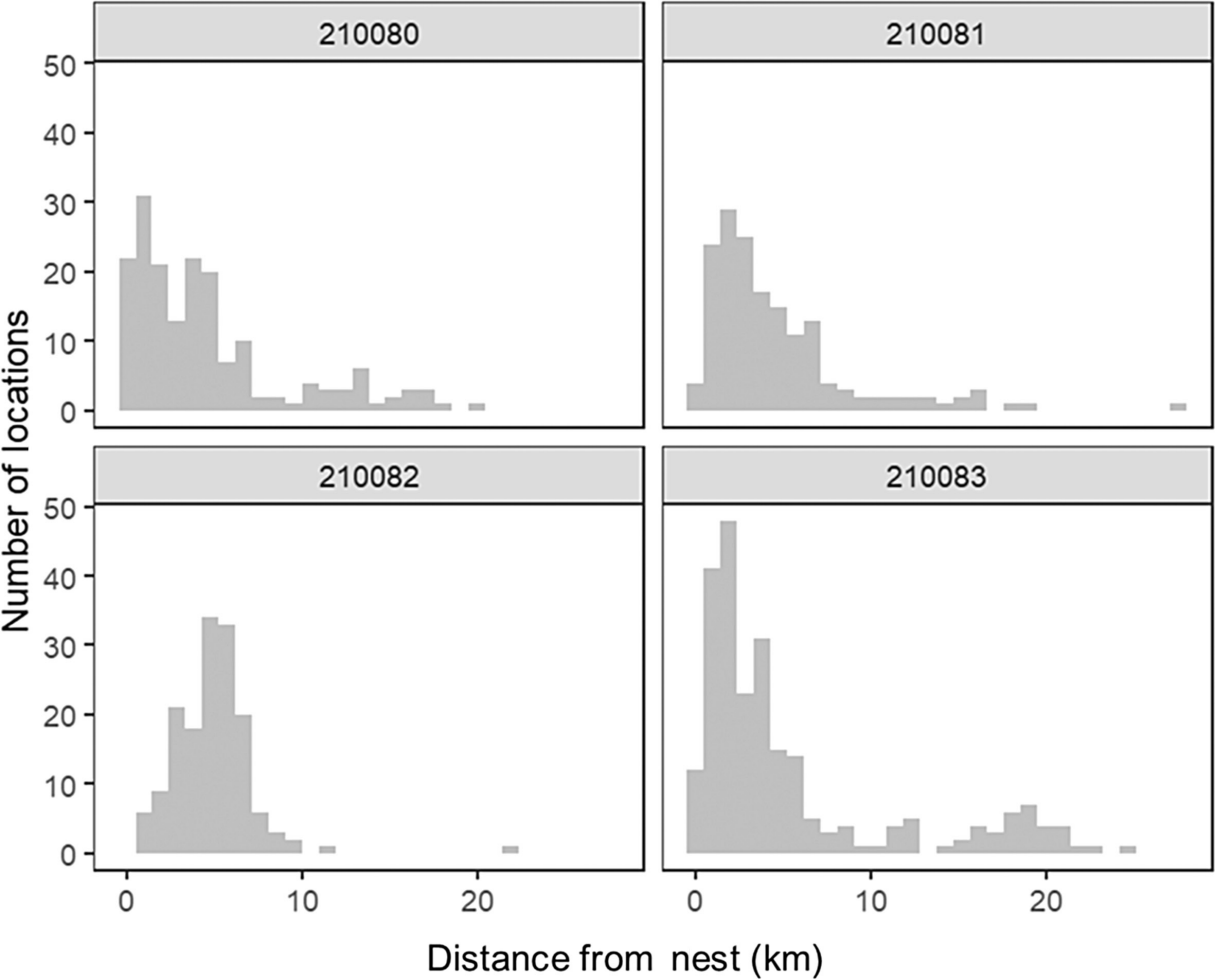
Histograms of the distance from the nest (km) for locations collected for Black Terns during the breeding season. Each panel represents one individual, 210080, 210081, 210082 and 210083.

During the breeding season, individuals moved as far as 35 km from the nest on foraging trips (Figure 3). Mean distance of each point from the known nest location was 8.85 ± 5.27 km, and 95% of locations were within 18.5 km of the nest.

### 3.3 Breeding season habitat selection

**FIGURE 4:**
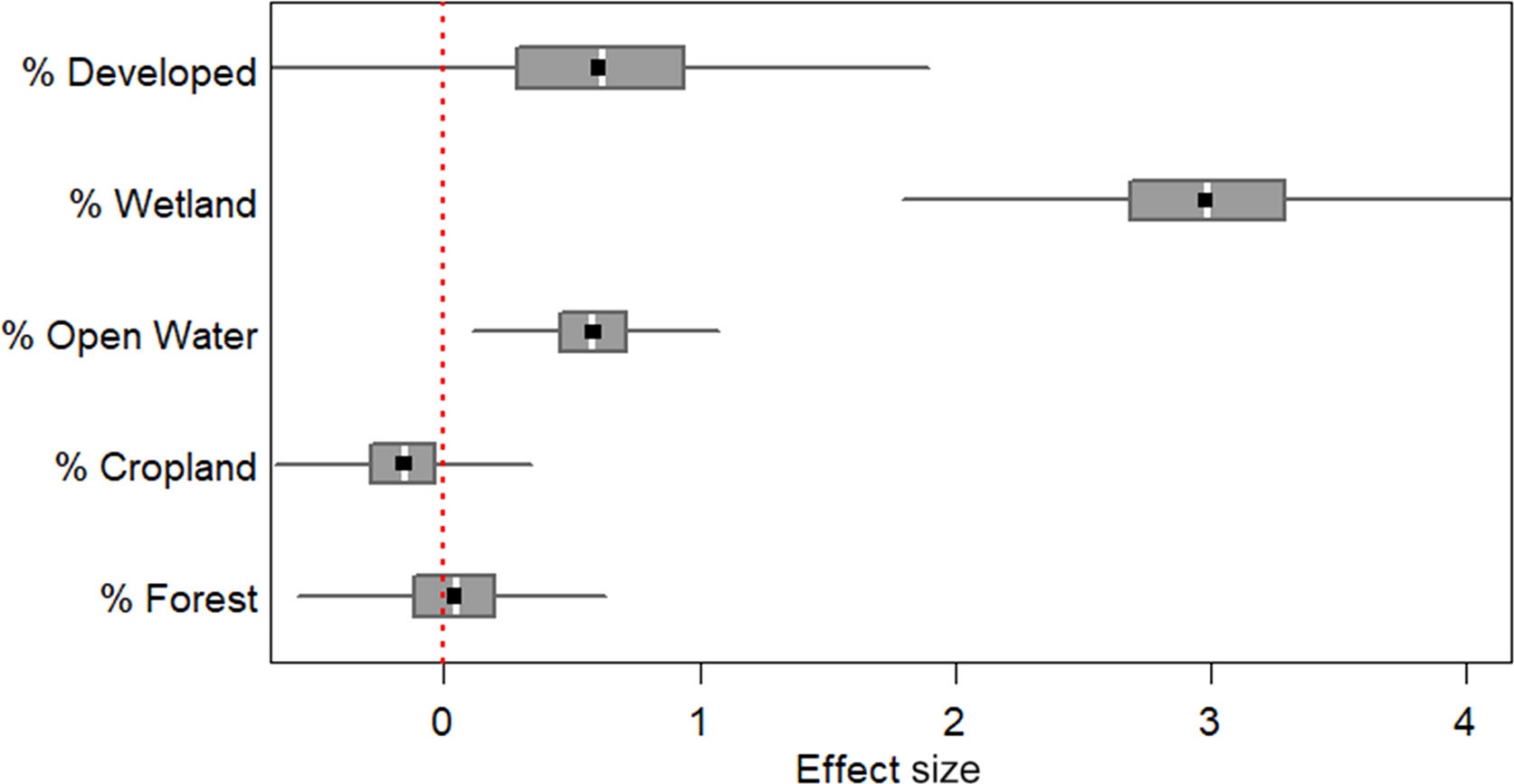
Posterior distributions for all land cover covariates for breeding areas, with effect size on the *x*‐axis. Each box shows the middle 50% credible interval of the posterior distribution, and the whiskers show the 95% credible interval. The white bar in the center of each box indicates the median, the black point in the center of each box indicates the mean and the dotted red line indicates 0 (i.e. no effect).

**FIGURE 5:**
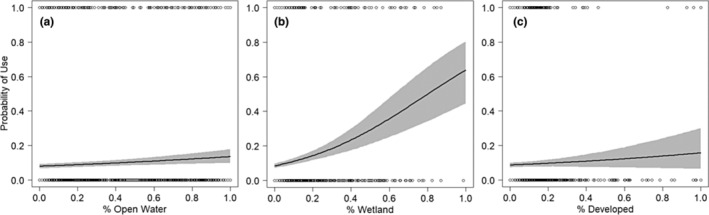
Prediction plots for covariates that explained substantial variation in probability of use for breeding areas: (a) open water, (b) wetland and (c) developed. The black solid lines show the mean, and the grey ribbon shows the 95% credible intervals. We considered an effect as explaining substantial variation in a response if more than 85% of posterior samples were above or below 0 (see text). Points show the raw data values for used (1) and unused (0) points.

Considering an effect as explaining substantial variation in a response if more than 85% of posterior samples were above or below zero, our analysis of habitat selection showed that variation in probability of use was not substantially explained by percent forest (*β* = −0.42, 95% CI [−0.42, 0.51], *p* = .56). There was a tendency for probability of use to be negatively related to percent cropland, although only 80% of the posterior samples were below zero (*β* = −0.16, 95% CI [−0.53, 0.24], *p* = .80). However, open water (*β* = 0.58, 95% CI [0.22, 0.98], *p* = 1.00), wetland (*β* = 2.98, 95% CI [2.16, 3.82], *p* = 1.00) and developed (*β* = 0.61, 95% CI [−0.34, 1.54], *p* = .89) all showed positive relationships with probability of use and explained substantial variation in probability of use (Figures 4 and 5). Results of the models run on each individual separately were generally consistent with the results based on all individuals pooled together when considering the direction of selection and proportion of posterior samples above or below 0 (but note that effect sizes cannot be compared between separate models; see Appendix S2). However, notably, 210082 showed that while developed land cover explained substantial variation in probability of use, the relationship was negative for this individual (*β* = −3.63, 95% CI [−7.48, −0.40], *p* = .99), while it was positive for all others and all in combination.

### 3.4 Habitat composition in breeding and stopover areas

Habitat composition differed somewhat between breeding and stopover locations, likely due to regional differences in climate, topography and land use (Table 1). Notably, approximate locations used during breeding had a greater proportion of forest cover (0.18 ± 0.01) and less grassland cover (0.07 ± 0.01) compared to locations used during stopover (0.00 ± 0.00 and 0.22 ± 0.02), whereas both breeding and stopover locations had similar proportions of wetland (0.04 ± 0.00 for breeding and 0.04 ± 0.01 for stopover) and developed land cover (0.03 ± 0.00 for breeding and 0.02 ± 0.00 for stopover). Overall, open water and cropland were the most prevalent habitat types surrounding used locations, likely due to their high prevalence on the landscape.

**TABLE 1:** Proportion of land cover (mean and standard error) within 150 m of locations in breeding (*n* = 700) and stopover (*n* = 189) of four individual Black Terns.

Land cover type	Breeding	Stopover
Forest	0.18 ± 0.01	0.00 ± 0.00
Wetland	0.04 ± 0.00	0.04 ± 0.01
Open water	0.26 ± 0.02	0.22 ± 0.03
Cropland	0.38 ± 0.02	0.50 ± 0.03
Grassland	0.07 ± 0.01	0.22 ± 0.02
Developed	0.03 ± 0.00	0.02 ± 0.00

## 4 DISCUSSION

We conducted the first‐ever satellite tracking of Black Terns and discovered that breeding home ranges were extensive (mean 283.7 km^2^) and distances travelled from nesting locations substantially greater (up to 35 km; mean 8.9 ± 2.3 km) than previously thought (2.4 ± 1.2 km; Mosher, 1986). While wetland and open water made up relatively small proportions of habitat composition in home ranges, they were the cover types most selected by terns during breeding, and individuals showed a tendency to avoid cropland. Interestingly, individuals showed positive selection for developed areas. All four tracked individuals made their first post‐breeding stopover within a relatively restricted area in North and South Dakota, although we did not have sufficient data to examine habitat selection during stopover. Despite a small sample size and limited precision of location data (150–1500 m), our results provide novel preliminary insights into area and habitat needs of this declining waterbird, with implications for conservation planning for the species, particularly within agriculturally dominated landscapes.

Studies of Black Terns have frequently made reference to typical foraging distances of ~2–5 km (Heath et al., 2020; Mosher, 1986) and some have based their selection of spatial scale for habitat association analyses on these values (Steen & Powell, 2012; Wyman & Cuthbert, 2016). But here we show that the species may have much larger area requirements during breeding than was previously thought. Our finding of mean home range size of 283.7 km^2^ (range 36.3–411.2 km^2^) corresponds somewhat, although is still larger, than the pre‐determined 25.7‐km^2^ grid cells that were used by Naugle et al. (1999, 2000) to summarize habitat composition across South Dakota; those studies concluded that in addition to the influence of local‐scale factors, black terns most often occurred in high‐density wetland complexes. Recent work on California spotted owls (*Strix occidentalis occidentalis*) using high‐resolution GPS tracking similarly noted that owls use more expansive areas during breeding than previously thought, and that many of these areas are outside U.S.D.A. Forest Service designated ‘Protected Activity Centers’ for this vulnerable subspecies, which is currently under review for federal listing (Blakey et al., 2019). Our results raise the question as to why terns are travelling such large distances from their colonies during the breeding season.

It has previously been noted that adult terns provision their chicks mostly with insects, which are often captured within ~500 m from the nest (Heath et al., 2020), and thus the large distances they travel may be to locate higher quality prey (i.e. small fish) to feed themselves. It has also been suggested that one parent may concentrate on fish (which are fed to chicks at a lower rate relative to other prey) and the other on insects, and that the former is often the male (Goodwin, 1960). This suggestion aligns with our results, given that all four individuals tracked in our study were males. Future studies should make use of tracking devices to compare male versus female foraging distances and home range sizes, although as noted previously, mass of tracking devices remains a limiting factor. It is also possible that differences in foraging distance we report compared to earlier work could be partly driven by differences in habitat between study areas. Our breeding study area in central Saskatchewan occurred at the northern edge of the PPR, a region heavily dominated by cropland interspersed with large lakes and smaller wetlands, just south of where the prairies transition into the boreal forest. Black Terns in this region may need to travel large distances to cross agricultural lands between patches of suitable wetland foraging habitat, and many of the smaller ‘pothole’ wetlands may not contain fish (Heath et al., 2020). In contrast, Mosher (1986) conducted studies within a large (6800 ha) wildlife management area in southeastern British Columbia. Here water levels on the marshes were regulated and periodically drained to maintain favourable vegetation conditions for wildlife (Mosher, 1986). Given a high‐density of presumably suitable habitat, adults may not have needed to travel as far to forage, especially for fish. Nonetheless, our results have important implications for habitat association studies conducted across the species range and highlight the need to consider local and regional habitat composition and heterogeneity when selecting the scale at which to evaluate habitat needs.

Not surprisingly for this wetland‐dependent species, probability of use within breeding home ranges was positively associated with percent wetland and open water. While we did not have sufficient data to examine habitat selection during stopover, the habitat composition at used locations during breeding and stopover was generally similar for most land cover types, although locations used during breeding had a higher proportion of open water and forest and less grassland. This could be indicative of fewer large lakes and waterbodies and less forest cover at the stopover locations in the Dakotas as compared to the wetland‐rich northern PPR. Interestingly, within their breeding home ranges terns apparently selected for developed areas , as defined by the Commission for Environmental Cooperation 30‐m resolution land cover classes. We interpret this result as evidence that humans and Black Terns are selecting for the same types of habitat features in our breeding study area, namely shorelines. In this region, there is relatively light development of shorelines that includes cottages, beaches and boat launches. Indeed, one of our study colonies is within a fringing marsh directly adjacent to a small beach and boat launch on a larger lake. This result implies that terns are able to tolerate the light degree of development in this area which could be due in part to their tendency to form generally small, loosely packed breeding colonies within emergent vegetation which may be difficult to for humans to enter and which prevents flooding from boat wash (Heath et al., 2020). This is in contrast to some other species of colonial gulls and terns, which may form larger, denser colonies which are easier to access and could be more sensitive to human disturbance (Carney & Syeman, 1999; but see Nisbet, 2000). On the other hand, there was some evidence for avoidance of cropland, as has been found in other studies where Black Terns avoided wetlands within landscapes where more than 50% of upland habitat was tilled (Naugle et al., 1999, 2000), although this effect was marginally significant in our case. Taken together, our results support those of other studies indicating the need for sufficient wetlands and waterbodies on the landscape to maintain avian biodiversity within agriculturally dominated areas (Berzins et al., 2022; Davies et al., 2016).

While we uncovered some interesting patterns with respect to home ranges, foraging distance and habitat selection that were previously undocumented for this species, our sample size was small and comprised only larger males. It is possible that findings would differ for smaller birds or females; it is also possible that the tags themselves affected individual behaviour (Bodey et al., 2018). However, we suspect any tag effects may have resulted in individuals travelling shorter distances on foraging trips if anything, thus indicating that the large distances we uncovered could be minimum estimates. It should also be noted that it is difficult to make strong inferences about fine‐scale habitat selection given the poor precision of Argos location data. There was a lot of uncertainty in our location estimates, which may have more strongly limited inference in a more heterogeneous landscape than in our breeding study area, where there were large expanses of land with relatively homogeneous land cover types (e.g. cropland). Indeed, habitat heterogeneity relative to the size of stopover areas and imprecision of location data, along with smaller sample sizes of locations, contributed to our lack of confidence in evaluating habitat selection in the stopover areas. Until smaller, high‐precision transmitting tags are available, further studies using manual radio telemetry or the deployment and retrieval of GPS logging tags within a breeding season could be alternative methods to increase sample size for individual tracking and habitat selection studies in Black Terns.

## AUTHOR CONTRIBUTIONS

**Ann E. McKellar:** Conceptualization (lead); funding acquisition (lead); investigation (lead); methodology (equal); project administration (lead); resources (lead); writing – original draft (lead); writing – review and editing (equal). **Sarah J. Clements:** Data curation (lead); formal analysis (lead); investigation (supporting); methodology (equal); visualization (lead); writing – original draft (supporting); writing – review and editing (equal).

## CONFLICT OF INTEREST STATEMENT

The authors declare that there is no conflict of interest regarding the publication of this article.

## Supporting information


Appendix S1‐S2

## Data Availability

Code used to run the JAGS model is provided in the Appendix S1 and S2. Tracking data are archived on Movebank (Study ID #1832505469) and are publicly available.

## References

[ece310716-bib-0001] Anderson, C. M. , Gilchrist, H. G. , Ronconi, R. A. , Shlepr, K. R. , Clark, D. E. , Weseloh, D. V. C. , Robertson, G. J. , & Mallory, M. L. (2019). Winter home range and habitat selection differs among breeding populations of herring gulls in eastern North America. Movement Ecology, 7, 8. 30891245 10.1186/s40462-019-0152-x PMC6404351

[ece310716-bib-0002] Batt, B. D. J. , Anderson, M. G. , Anderson, C. D. , & Casewell, F. D. (1989). The use of prairie potholes by north American ducks. In A. G. van der Valk (Ed.), Northern prairie wetlands (pp. 204–227). Iowa State University Press.

[ece310716-bib-0003] Berzins, L. L. , Morrissey, C. A. , Howerter, D. W. , & Clark, R. G. (2022). Conserving wetlands in agroecosystems can sustain aerial insectivore productivity and survival. Canadian Journal of Zoology, 100, 617–629.

[ece310716-bib-0004] Beyersbergen, G. W. , Niemuth, N. D. , & Norton, M. R. (2004). Northern prairie and parkland waterbird conservation plan. Prairie Pothole Joint Venture.

[ece310716-bib-0005] Blakey, R. V. , Siegel, R. B. , Webb, E. B. , Dillingham, C. P. , Bauer, R. L. , Johnson, M. , & Kesler, D. C. (2019). Space use, forays, and habitat selection by California spotted owls (*Strix occidentalis occidentalis*) during the breeding season: New insights from high resolution GPS tracking. Forest Ecology and Management, 432, 912–922.

[ece310716-bib-0006] Bodey, T. W. , Cleasby, I. R. , Bell, F. , Parr, N. , Schultz, A. , Votier, S. C. , & Bearhop, S. (2018). A phylogenetically controlled meta‐analysis of biologging device effects on birds: Deleterious effects and a call for more standardized reporting of study data. Methods in Ecology and Evolution, 9, 946–955.

[ece310716-bib-0007] Borrmann, R. M. , Phillips, R. A. , Clay, T. A. , & Garthe, S. (2019). High foraging site fidelity and spatial segregation among individual great black‐backed gulls. Journal of Avian Biology, 50, 1–10.

[ece310716-bib-0008] Bracey, A. M. , Etterson, M. A. , Strand, F. C. , Matteson, S. W. , Niemi, G. J. , Cuthbert, F. J. , & Hoffman, J. C. (2020). Foraging ecology differentiates life stages and mercury exposure in common terns (*Sterna hirundo*). Integrated Environmental Assessment and Management, 17, 398–410. 32930480 10.1002/ieam.4341 PMC8108127

[ece310716-bib-0009] Brooks, S. P. , & Gelman, A. (1998). General methods for monitoring convergence of iterative simulations. Journal of Computational and Graphical Statistics, 7, 434–455.

[ece310716-bib-0010] Brown, M. , & Dinsmore, J. J. (1986). Implications of marsh size and isolation for marsh bird management. Journal of Wildlife Management, 50, 392–397.

[ece310716-bib-0011] Canada Centre for Remote Sensing (CCRS), Canada Centre for Mapping and Earth Observation (CCMEO), Natural Resources Canada (NRCan), Comisión Nacional para el Conocimiento y Uso de la Biodiversidad (CONABIO), Comisión Nacional Forestal (CONAFOR), Instituto Nacional de Estadística y Geografía (INEGI), & U.S. Geological Survey (USGS) . (2020). 2015 Land Cover of North America at 30 meters. http://www.cec.org/north‐american‐environmental‐atlas/land‐cover‐30m‐2015‐landsat‐and‐rapideye/

[ece310716-bib-0012] Cañadas, A. , Sagarminaga, R. , De Stephanis, R. , Urquiola, E. , & Hammond, P. S. (2005). Habitat preference modelling as a conservation tool: Proposals for marine protected areas for cetaceans in southern Spanish waters. Aquatic Conservation, 15, 495–521.

[ece310716-bib-0013] Carney, K. M. , & Syeman, W. J. (1999). A review of human disturbance effects on nesting colonial waterbirds. Waterbirds, 22, 68–79.

[ece310716-bib-0014] Chetkiewicz, C.‐L. B. , & Boyce, M. S. (2009). Use of resource selection functions to identify conservation corridors. Journal of Applied Ecology, 46, 1036–1047.

[ece310716-bib-0015] Davies, S. R. , Sayer, C. D. , Greaves, H. , Siriwardena, G. M. , & Axmacher, J. C. (2016). A new role for pond management in farmland bird conservation. Agricultura, Ecosystems & Environment, 233, 179–191.

[ece310716-bib-0016] Doherty, K. E. , Howerter, D. W. , Devries, J. H. , & Walker, J. (2018). Prairie pothole region of North America. In C. M. Finlayson , G. R. Milton , R. C. Prentice , & N. C. Davidson (Eds.), The wetland book II: Distribution, description, and conservation (pp. 1–10). Springer.

[ece310716-bib-0017] Douglas, D. C. , Weinzierl, R. , Davidson, S. C. , Kays, R. , Wikelski, M. , & Bohrer, G. (2012). Moderating Argos location errors in animal tracking data. Methods in Ecology and Evolution, 3, 999–1007.

[ece310716-bib-0018] Fleming, C. H. , Fagan, W. F. , Mueller, T. , Olson, K. A. , Leimgruber, P. , & Calabrese, J. M. (2015). Rigorous home range estimation with movement data: A new autocorrelated kernel density estimator. Ecology, 96, 1182–1188. 26236833 10.1890/14-2010.1

[ece310716-bib-0019] Goodwin, R. E. (1960). A study of the ethology of the black tern, Chlidonias Niger surinamensis (Gmelin). PhD Thesis. Cornell University.

[ece310716-bib-0020] Heath, S. R. , Dunn, E. H. , & Agro, D. J. (2020). Black tern (*Chlidonias Niger*), version 1.0. In S. M. Billerman (Ed.), Birds of the world. Cornell Lab of Ornithology. 10.2173/bow.blkter.01

[ece310716-bib-0021] Kellner, K. (2019). jagsUI: A Wrapper Around ‘rjags’ to Streamline ‘JAGS’ Analyses. R Package Version 1.1.

[ece310716-bib-0022] Kranstauber, B. , Cameron, A. , Weinzerl, R. , Fountain, T. , Tilak, S. , Wikelski, M. , & Kays, R. (2011). The Movebank data model for animal tracking. Environmental Modelling & Software, 26, 834–835.

[ece310716-bib-0023] Kremen, C. , & Merenlender, A. M. (2018). Landscapes that work for biodiversity and people. Science, 362, 304. 10.1126/science.aau6020 30337381

[ece310716-bib-0024] Mallory, M. L. , & Gilbert, C. D. (2008). Leg‐loop harness design for attaching external transmitters to seabirds. Marine Ornithology, 36, 183–188.

[ece310716-bib-0025] Mayor, S. J. , Schneider, D. C. , Schaefer, J. A. , & Mahoney, S. P. (2009). Habitat selection at multiple scales. Ecoscience, 16, 238–247.

[ece310716-bib-0026] Mosher, B. C. (1986). Factors influencing reproductive success and nesting strategies in black terns. Ph.D. Dissertation. Simon Fraser University.

[ece310716-bib-0027] Mosloff, A. R. , Weegman, M. D. , Thompson, F. R. , & Thompson, T. R. (2021). Northern bobwhite select for shrubby thickets interspersed in grasslands during fall and winter. PLoS One, 16, e0255298. 34407114 10.1371/journal.pone.0255298 PMC8372937

[ece310716-bib-0028] Naugle, D. E. , Higgins, K. F. , Estey, M. E. , Johnson, R. R. , & Nusser, S. M. (2000). Local and landscape‐level factors influencing black tern habitat suitability. Journal of Wildlife Management, 64, 253–260.

[ece310716-bib-0029] Naugle, D. E. , Higgins, K. F. , Nusser, S. M. , & Johnson, W. C. (1999). Scale‐dependent habitat use in three species of prairie wetland birds. Landscape Ecology, 14, 267–276.

[ece310716-bib-0030] Newbold, T. , Hudson, L. N. , Hill, S. L. L. , Contu, S. , Lysenko, I. , Senior, R. A. , Börger, L. , Bennett, D. J. , Choimes, A. , Collen, B. , Day, J. , De Palma, A. , Díaz, S. , Echeverria‐Londoño, S. , Edgar, M. J. , Feldman, A. , Garon, M. , Harrison, M. L. K. , Alhusseini, T. , … Purvis, A. (2015). Global effects of land use on local terrestrial biodiversity. Nature, 520, 45–50. 25832402 10.1038/nature14324

[ece310716-bib-0031] Nisbet, I. C. T. (2000). Disturbance, habituation, and management of waterbird colonies. Waterbirds, 23, 312–332.

[ece310716-bib-0032] Perfecto, I. , Vandermeer, J. , & Wright, A. (2009). Nature's matrix: Linking agriculture. Conservation and Food Sovereignty.

[ece310716-bib-0033] Plummer, M. (2003). JAGS: A program for analysis of Bayesian graphical models using Gibbs sampling. *Proceedings of the 3rd international workshop on distributed statistical computing*, vol. 124 (pp. 1–10).

[ece310716-bib-0034] Pringle, R. M. (2017). Upgrading protected areas to conserve wild biodiversity. Nature, 546, 91–99. 28569807 10.1038/nature22902

[ece310716-bib-0046] R Core Team . (2022). R: A language and environment for statistical computing. R Foundation for Statistical Computing. https://www.R‐project.org/

[ece310716-bib-0035] Shealer, D. A. , & Alexander, M. J. (2013). Use of aerial imagery to assess habitat suitability and predict site occupancy for a declining wetland‐dependent bird. Wetlands Ecology and Management, 21, 289–296.

[ece310716-bib-0036] Shephard, N. G. , Reudink, M. W. , & McKellar, A. E. (2023). Assessing black tern (*Chlidonias Niger*) habitat associations in Saskatchewan using aerial imagery. Waterbirds, 45, 247–258.

[ece310716-bib-0037] Shephard, N. G. , Szczys, P. , Moore, D. J. , Reudink, M. W. , Costa, J. N. , Bracey, A. M. , Lisovski, S. , & McKellar, A. E. (2023). Weak genetic structure, shared nonbreeding areas, and extensive movement in a declining waterbird. Ornithological Applications, 125, duac053. 10.1093/ornithapp/duac053

[ece310716-bib-0038] Signer, J. , Fieberg, J. , & Avgar, T. (2019). Animal movement tools (amt): R package for managing tracking data and conducting habitat selection analyses. Ecology and Evolution, 9, 880–890. 30766677 10.1002/ece3.4823 PMC6362447

[ece310716-bib-0039] Soriano‐Redondo, A. , Acácio, M. , Franco, A. M. , Herlander Martins, B. , Moreira, F. , Rogerson, K. , & Catry, I. (2020). Testing alternative methods for estimation of bird migration phenology from GPS tracking data. Ibis, 162(2), 581–588.

[ece310716-bib-0040] Steen, V. A. , & Powell, A. N. (2012). Wetland selection by breeding and foraging black terns in the prairie pothole region of the United States. The Condor, 114, 155–165.

[ece310716-bib-0041] Tonra, C. M. , Hallworth, M. T. , Boves, T. H. , Reese, J. , Bulluck, L. P. , Johnson, M. , Viverette, C. , Percy, K. , Ames, E. M. , Matthews, A. , Slevin, M. C. , Wilson, R. R. , & Johnson, E. I. (2019). Concentration of a widespread breeding population in a few critically important nonbreeding areas: Migratory connectivity in the prothonotary warbler. The Condor, 121, duz019.

[ece310716-bib-0042] Tscharntke, T. , Clough, Y. , Wanger, T. C. , Jackson, L. , Motzke, I. , Perfecto, I. , Vandermeer, J. , & Whitbread, A. (2012). Global food security, biodiversity conservation and the future of agricultural intensification. Biological Conservation, 151, 53–59.

[ece310716-bib-0043] Venter, O. , Brodeur, N. N. , Nemiroff, L. , Belland, B. , Dolinsek, I. J. , & Grant, J. W. A. (2006). Threats to endangered species in Canada. Bioscience, 56, 903–910.

[ece310716-bib-0044] Watson, J. E. M. , Dudley, N. , Segan, D. B. , & Hockings, M. (2014). The performance and potential of protected areas. Nature, 515, 67–73. 25373676 10.1038/nature13947

[ece310716-bib-0045] Wyman, K. E. , & Cuthbert, F. J. (2016). Validation of landscape suitability indices for black terns (*Chlidonias Niger*) in the U.S. Great Lakes region. The Condor, 118, 613–623.

