# Supplementary material for: First‐ever satellite tracking of Black Terns (Chlidonias niger): Insights into home range and habitat selection
Source: Ecol Evol. 2023 Nov 15;13(11):e10716. doi: 10.1002/ece3.10716 (PMC10651349; doi:10.1002/ece3.10716)
Supplement: Supplementary file 1 — Appendix S1‐S2 [file ECE3-13-e10716-s001.docx]

**Appendix**

Supporting information for: McKellar, A.E. and Clements, S.J. First-ever satellite tracking of Black Terns (*Chlidonias niger*): insights into home range and habitat selection. Ecology & Evolution.

**Appendix S1.** Code for Jags Model

# Model for Home range and habitat selection of Black Terns

# AE McKellar, SJ Clements

## Load Packages and Data

#This file was created by using the amt package to generate home ranges and

#sample random points within the home ranges, then extracting the proportion

#of each landcover type within a 150m buffer of each point.

#The spatial data set can be found here:

# http://www.cec.org/north-american-environmental-atlas/land-cover-30m-2015-landsat-and-rapideye/

library(jagsUI)

library(boot)

library(amt)

# data

dt <- read.csv('G:\\My Drive\\tern\\tern_loc_breed_raw_101322.csv')

# provide this data file on github, with link?

## Reclassify Landcover and Prepare Data for Model

# landcover classes

#Value 1, Temperate or sub-polar needleleaf forest, RGB 0 61 0;

#Value 2, Sub-polar taiga needleleaf forest, RGB 148 156 112;

#Value 3, Tropical or sub-tropical broadleaf evergreen forest, RGB 0 99 0;

#Value 4, Tropical or sub-tropical broadleaf deciduous forest, RGB 30 171 5;

#Value 5, Temperate or sub-polar broadleaf deciduous forest, RGB 20 140 61;

#Value 6, Mixed forest, RGB 92 117 43;

#Value 7, Tropical or sub-tropical shrubland, RGB 179 158 43;

#Value 8, Temperate or sub-polar shrubland, RGB 179 138 51;

#Value 9, Tropical or sub-tropical grassland, RGB 232 220 94;

#Value 10, Temperate or sub-polar grassland, RGB 225 207 138;

#Value 11, Sub-polar or polar shrubland-lichen-moss, RGB 156 117 84;

#Value 12, Sub-polar or polar grassland-lichen-moss, RGB 186 212 143;

#Value 13, Sub-polar or polar barren-lichen-moss, RGB 64 138 112;

#Value 14, Wetland, RGB 107 163 138;

#Value 15, Cropland, RGB 230 174 102;

#Value 16, Barren lands, RGB 168 171 174;

#Value 17, Urban, RGB 220 33 38;

#Value 18, Water, RGB 76 112 163;

#Value 19, Snow and Ice, RGB 255 250 255.

ids <- as.numeric(as.factor(dt$id))

forest <- dt$c1+dt$c5+dt$c6

grass <- dt$c10

wetland <- dt$c14

crop <- dt$c15

developed <- dt$c17

water <- dt$c18

nest_dist <- dt$nest_dist

case <- as.numeric(as.factor(dt$case_)) # 1= unused 2=used

case[which(case==1)] <- 0 # change unused to 0

case[which(case==2)] <- 1 # change used to 1

# data frame for all data

rsf_all <- data.frame(case=as.factor(case), id=dt$id, forest=forest, grass=grass, wetland=wetland, crop=crop, developed=developed,

water=water)

cor(rsf_all[2:ncol(rsf_all)]) # check if things are correlated

# Jags model (requires jags to be installed on the computer)

# iterations, thin, burn in, chains

ni <- 5000

nt <- 5

nb <- 2000

nc <- 3

sink("m2.jags")

cat("

# likelihood

model{

for (i in 1:N){

case[i] ~ dbern(p[i])

logit(p[i]) <- fr*forest[i] + cr*crop[i]+ wl*wetland[i]+ wt*water[i] +

dv*developed[i] + id[individual[i]] + alpha

}

# priors

alpha ~ dnorm(0,0.001)

# if reference condition for ID

id[1] <- 0

for (i in 2:4){

id[i] ~ dnorm(0,0.001)

}

fr ~ dnorm(0,0.001)

cr ~ dnorm(0,0.001)

wl ~ dnorm(0,0.001)

wt ~ dnorm(0,0.001)

dv ~ dnorm(0,0.001)

}

",fill = TRUE)

sink()

# Bundle data

jags.data <- list(case=case, forest=forest, individual=ids, grass=grass,

wetland=wetland, developed=developed, crop=crop, water=water,

N = length(case))

# Initial values

inits <- function (){list(alpha = 0, id=c(NA, rep(0, 3)))}

# Parameters monitored

parameters <- c("fr","cr" ,"wt", "wl", "dv", "id","alpha")

# Call jags from R

m2 <- jags(data=jags.data, inits=inits, parameters.to.save=parameters, model.file="m2.jags",

n.chains = nc, n.thin = nt, n.iter = ni, n.burnin = nb, parallel=T)

print(m2)

jagsUI::traceplot(m2)

**Appendix S2.** Tables of effect sizes for individual Black Tern probability of use models, where fr, cr, wl, wt, and dv are the percent forest, percent cropland, percent wetland, percent open water, and percent developed, respectively.

**Table S2.1**. Individual 210080.

|  | **mean** | **sd** | **2.50%** | **25%** | **50%** | **75%** | **97.50%** | **Rhat** | **n.eff** | **overlap0** | **f** |
| --- | --- | --- | --- | --- | --- | --- | --- | --- | --- | --- | --- |
| **fr** | -0.06 | 0.92 | -1.92 | -0.68 | -0.02 | 0.59 | 1.64 | 1.00 | 1800.00 | 1.00 | 0.51 |
| **cr** | 0.12 | 0.39 | -0.60 | -0.15 | 0.10 | 0.37 | 0.92 | 1.00 | 622.00 | 1.00 | 0.62 |
| **wt** | 1.09 | 0.39 | 0.36 | 0.82 | 1.07 | 1.36 | 1.90 | 1.00 | 821.00 | 0.00 | 1.00 |
| **wl** | 5.51 | 1.03 | 3.56 | 4.82 | 5.48 | 6.16 | 7.55 | 1.00 | 1800.00 | 0.00 | 1.00 |
| **dv** | 1.44 | 0.83 | -0.25 | 0.89 | 1.45 | 2.01 | 3.01 | 1.00 | 533.00 | 1.00 | 0.95 |
| **alpha** | -2.84 | 0.35 | -3.55 | -3.06 | -2.82 | -2.60 | -2.19 | 1.00 | 535.00 | 0.00 | 1.00 |
| **deviance** | 996.91 | 3.53 | 992.06 | 994.33 | 996.27 | 998.72 | 1005.67 | 1.00 | 807.00 | 0.00 | 1.00 |

**Table S2.2**. Individual 210081.

|  | **mean** | **sd** | **2.50%** | **25%** | **50%** | **75%** | **97.50%** | **Rhat** | **n.eff** | **overlap0** | **f** |
| --- | --- | --- | --- | --- | --- | --- | --- | --- | --- | --- | --- |
| **fr** | -1.43 | 0.62 | -2.68 | -1.83 | -1.40 | -1.02 | -0.25 | 1.00 | 1800.00 | 0.00 | 0.99 |
| **cr** | -0.85 | 0.32 | -1.47 | -1.06 | -0.85 | -0.64 | -0.20 | 1.00 | 1800.00 | 0.00 | 1.00 |
| **wt** | 0.24 | 0.33 | -0.41 | 0.02 | 0.25 | 0.47 | 0.89 | 1.00 | 1149.00 | 1.00 | 0.78 |
| **wl** | 2.10 | 1.11 | -0.24 | 1.41 | 2.15 | 2.86 | 4.10 | 1.00 | 1800.00 | 1.00 | 0.97 |
| **dv** | 0.62 | 0.85 | -1.04 | 0.05 | 0.63 | 1.21 | 2.25 | 1.00 | 1800.00 | 1.00 | 0.77 |
| **alpha** | -1.89 | 0.27 | -2.44 | -2.07 | -1.87 | -1.70 | -1.35 | 1.00 | 1800.00 | 0.00 | 1.00 |
| **deviance** | 1042.23 | 3.50 | 1037.42 | 1039.60 | 1041.61 | 1044.17 | 1050.39 | 1.00 | 1340.00 | 0.00 | 1.00 |

**Table S2.3**. Individual 210082.

|  | **mean** | **sd** | **2.50%** | **25%** | **50%** | **75%** | **97.50%** | **Rhat** | **n.eff** | **overlap0** | **f** |
| --- | --- | --- | --- | --- | --- | --- | --- | --- | --- | --- | --- |
| **fr** | -0.24 | 0.87 | -1.96 | -0.84 | -0.25 | 0.34 | 1.45 | 1.00 | 949.00 | 1.00 | 0.60 |
| **cr** | -0.21 | 0.43 | -1.03 | -0.50 | -0.23 | 0.08 | 0.66 | 1.00 | 830.00 | 1.00 | 0.69 |
| **wt** | 0.11 | 0.44 | -0.72 | -0.18 | 0.11 | 0.39 | 0.97 | 1.00 | 583.00 | 1.00 | 0.60 |
| **wl** | 2.52 | 0.67 | 1.27 | 2.04 | 2.53 | 2.97 | 3.83 | 1.00 | 1256.00 | 0.00 | 1.00 |
| **dv** | -3.63 | 1.75 | -7.48 | -4.69 | -3.52 | -2.46 | -0.40 | 1.01 | 340.00 | 0.00 | 0.99 |
| **alpha** | -2.28 | 0.40 | -3.10 | -2.54 | -2.27 | -2.02 | -1.54 | 1.00 | 658.00 | 0.00 | 1.00 |
| **deviance** | 958.21 | 3.55 | 953.53 | 955.64 | 957.47 | 960.06 | 966.70 | 1.00 | 1800.00 | 0.00 | 1.00 |

**Table S2.4**. Individual 210083.

|  | **mean** | **sd** | **2.50%** | **25%** | **50%** | **75%** | **97.50%** | **Rhat** | **n.eff** | **overlap0** | **f** |
| --- | --- | --- | --- | --- | --- | --- | --- | --- | --- | --- | --- |
| **fr** | 1.21 | 0.52 | 0.24 | 0.86 | 1.18 | 1.55 | 2.30 | 1.00 | 756.00 | 0.00 | 1.00 |
| **cr** | 0.93 | 0.51 | -0.02 | 0.58 | 0.89 | 1.27 | 1.98 | 1.00 | 1036.00 | 1.00 | 0.97 |
| **wt** | 1.35 | 0.51 | 0.44 | 0.99 | 1.32 | 1.67 | 2.44 | 1.00 | 743.00 | 0.00 | 1.00 |
| **wl** | -12.03 | 8.85 | -33.28 | -16.75 | -10.63 | -5.45 | 0.65 | 1.01 | 892.00 | 1.00 | 0.96 |
| **dv** | 3.17 | 1.22 | 0.81 | 2.34 | 3.16 | 4.00 | 5.59 | 1.00 | 796.00 | 0.00 | 1.00 |
| **alpha** | -3.42 | 0.49 | -4.44 | -3.75 | -3.39 | -3.09 | -2.54 | 1.00 | 748.00 | 0.00 | 1.00 |
| **deviance** | 1583.08 | 3.36 | 1578.48 | 1580.69 | 1582.50 | 1584.85 | 1591.59 | 1.00 | 1800.00 | 0.00 | 1.00 |
